# Moving Object Detection Using Scanning Camera on a High-Precision Intelligent Holder

**DOI:** 10.3390/s16101758

**Published:** 2016-10-21

**Authors:** Shuoyang Chen, Tingfa Xu, Daqun Li, Jizhou Zhang, Shenwang Jiang

**Affiliations:** 1 School of Optoelectronics, Image Engineering & Video Technology Lab, Beijing Institute of Technology, Beijing 100081, China; 2120140517@bit.edu.cn (S.C.); ldq_1221@163.com (D.L.); xiaomianzhou@126.com (J.Z.); jiangwenj02@163.com (S.J.); 2 Key Laboratory of Photoelectronic Imaging Technology and System, Ministry of Education of China, Beijing 100081, China

**Keywords:** moving object detection, background modeling, intelligent visual surveillance

## Abstract

During the process of moving object detection in an intelligent visual surveillance system, a scenario with complex background is sure to appear. The traditional methods, such as “frame difference” and “optical flow”, may not able to deal with the problem very well. In such scenarios, we use a modified algorithm to do the background modeling work. In this paper, we use edge detection to get an edge difference image just to enhance the ability of resistance illumination variation. Then we use a “multi-block temporal-analyzing LBP (Local Binary Pattern)” algorithm to do the segmentation. In the end, a connected component is used to locate the object. We also produce a hardware platform, the core of which consists of the DSP (Digital Signal Processor) and FPGA (Field Programmable Gate Array) platforms and the high-precision intelligent holder.

## 1. Introduction

Moving object detection is an important part of computer vision filed. All of the identification and tracking parts afterwards depend on a precise and robust detection result, which makes the moving object detection step a really important portion [1,2,3,4,5]. Moving object detection can be used in many scenarios, such as intelligent video surveillance, motion analysis, and human–machine interface applications, therefore deep research of this topic is meaningful [6,7,8,9,10].

Researchers usually employ some sensors to gain information for the processing system [11,12,13]. In this paper, a scanning moving object detection system is used with a scanning high definition camera as its video image sensor. High-definition camera is widely used in both indoor and outdoor video surveillance systems. We can get far more than one field of view since we made our video image sensor scan around with the support of the holder mentioned before. This means we can gain richer scenes information than just using a high-definition camera. In this paper, the main work we have done is detecting one or more moving objects from the video sequence gained by a high definition camera rotating around using an intelligent holder. Scenarios including tracking and positioning of fire, personnel and vehicles are all applicable.

A surveillance system can be defined as a technological tool that assists humans by providing an extended perception and reasoning capability about situations of interest that occur in the monitored environments [14]. Human perception and reasoning are constrained by the capabilities and limits of human senses and mind to simultaneously collect, process, and store limited data.

Visual surveillance research focuses on intelligent visual surveillance (IVS) in a wide area. Research trends in IVS can be divided largely into image interpretation and wide area surveillance control techniques. The goal of image interpretation is to extract high-level information of a visual event from a dynamic scene [15]. Image interpretation often includes motion detection, object recognition, tracking, and behavior understanding. In this paper, the function of our moving object detecting system (belonging to IVS) is to detect the moving object from a complex outdoor background.

We put a high-definition camera on a High-precision Intelligent Holder. The positional accuracy of the presetting point achieves 0.1°. Reliable repetitive precision is available for multiple times of access. It is specially designed for continuous operation. The worm and gear driving device and stepper motor ensure the long service life and zero-deflection operation. Minimum speed 0.01°/s and maximum speed 25°/s satisfy different requirements of long-range and short-range observation. The data echo of focal length of lens and holder angle is supported, which is convenient for the back end to carry out intelligent analysis. A stabilized gyro connector is provided at the holder, which, together with a gyro instrument, will ensure the stable image in the on-vehicle, on-ship and on-plane conditions. When the holder rotates around in a given rule, the camera can gain a complete image of these areas.

There are some traditionally and widely used algorithms including frame difference method, traditional background subtraction method, optical flow method and statistical learning method [16]. The previous two methods only work on a stationary background. However, our research is on a dynamic background video, which is gained by a camera, meaning those two methods cannot be used directly. In this paper, as we build a camera scanning system, an aspect we thought about is the real-time performance of this system, which means that the computing complexity of our method should not be too high. Now, with reference to the basic method above, we can find that both optical flow [17,18,19] and statistical learning method [20,21,22] have a large amount of calculation. The edge detection method does not cost much computation time. In addition, the LBP (Local Binary Pattern) algorithm we use has less computational complexity and does not have complex iterative process. Even combined with the edge detection process, our method could still meet the real-time requirement. In addition, real-time surveillance systems, especially in the outdoor condition, have a high background complexity; that is, when the object detection method proceeds, we should consider the background complexity. Considering the illumination variation of the complex background, we will use the edge detection and the LBP algorithm together to dilute the influence. As mentioned above, moving object detection is a key part of a computer vision system and provides initial information about the object; thus, we can extract as much information as possible. When obtaining the edge differencing image, we cannot get as much information as we need. To avoid this, we perform background modeling and background subtraction to obtain more data from the moving object that needs to be extracted from the video. After that, we bring the results together and generate the final result. To achieve the real-time requirement, we also did some optimization work on the program. Though our method does not have a very high frame frequency, the processing speed at 19–35 fps can still achieve the real-time requirement.

In view of the four methods above, which are based on a stationary background, the video sequence we get from our video image sensor has a dynamic background. The proposed method uses LBP operator and edge detection is processed to do the background modeling and moving object detection work. From the above analysis, we can find that the exiting algorithms have drawbacks on the scenario with illumination variations, dynamic background and video noise. Our method can solve these three problems well by using the LBP operator and the speed of this detection process will be improved.

In this paper, we first use edge detection to get an edge difference image. After that, we conduct the multi-block temporal-analyzing LBP method and combine edge detection to do the moving object detection work. To elaborate these, we will discuss the related work in Section 2. The moving object detection work is shown in Section 3 (including edge detection, background modeling and object locating. The experiment and analysis are presented in Section 4. Finally, Section 5 concludes the paper.

## 2. Related Work

There is an evaluation of background subtraction provided by Brutzer et al. [23]. In this section, we introduce some recent algorithms to do background modeling and moving object detection, which is the most related work to ours.

### 2.1. Intelligent Visual Surveillance

Intelligent visual surveillance improves conventional passive surveillance systems through automated object recognition and tracking, scene interpretation and indexing/retrieval of visual events. Visual surveillance techniques have initiated a wide variety of applications in access control, person specific identification, anomaly detection and alarming in academic community as well as industry and government [24]. Large research projects on visual surveillance have driven realization of practical visual surveillance systems. Successful visual surveillance systems such as the Visual Surveillance and Monitoring (VSAM) [25], the Annotated Digital Video for Intelligent Surveillance and Optimized Retrieval (ADVISOR) [26], and the Smart Surveillance System of IBM [27] have been developed by combining computer vision, system engineering, and communication techniques. Wang et al. [28] show an intelligent multi-camera video surveillance which emphasizes the connection and integration of different modules. Song et al. [29] present a comprehensive survey of recent research results to address the problems of intra-camera tracking, topological structure learning, target appearance modeling, and global activity understanding in sparse camera networks.

### 2.2. Moving Object Detection

Moving object detection and background modeling work always have to face these five major challenges: illumination changes, dynamic background, camouflage, bootstrapping and video noise. Most of the background modeling work starts around these challenges. In recent years, researchers explored some work on these problems. Cheng et al. [30] proposes a novel hybrid background subtraction technique to solve the bootstrapping problem. Huang et al. [31] have presented a novel motion detection approach based on radial basis function artificial neural networks to accurately detect moving objects not only in dynamic scenes but also in static scenes. Chen et al. [32] have presented an approach that is applicable not only in high bit-rate video streams but also in low bit-rate video streams. Cao et al. [33] have presented a unified framework for addressing the difficulties of dynamic background and irregular object movement, especially the one caused by irregular object movement. Zhang et al. [34] have proposed a new strategy to detect camouflaged moving objects which can identify the truly camouflaged areas. The approaches above all solved one or two problems from the aforementioned challenges. In this paper, the approach MB-TALBP (Multi-Block Temporal-Analyzing Local Binary Pattern) we present can solve three of the five challenges (which are illumination changes, dynamic background and video noise) together.

There are also some classic algorithms that play an important role in the moving object detection field. Stauffer et al. [35] have presented a background mixture model by a mixture of Gaussians and using an on-line approximation to update the model. The existing algorithm of Gaussian mixture background modeling [36,37,38] performs well in some cases. There are some new algorithms or improved algorithms that are based on the principles of Gaussian mixture algorithm. However, the Gaussian mixture algorithms also have some drawbacks, such as a relatively large amount of calculation, lack pace, and sensitive to illumination variation. The codebook algorithm [39,40,41], which has good results, is also sensitive to illumination variation. Wang et al. [42,43] have proposed moving target detection algorithm based on the adaptive statistical efficiency, the algorithm is called SACON (SAmple CONsensus). Calculating an estimated background model through each pixel sample consistency is simple, but it still has very strong performance. Comparing to the current algorithms, it can be seen that the algorithm has strong prospects. ViBe algorithm [44,45], which has fast speed and small amount of computation, is very good, but has a certain degree of noise robustness problem. Background modeling method based on color information [46,47,48], referred to as color algorithms, that decompose the difference of pixels’ Chromaticity and Brightness into differences in illumination has strong robustness, better results and faster speed, and, thus, can meet the basic requirements of real-time. Elgammal et al. [49,50] use kernel density estimation to establish a non-parametric background model. For each pixel, in order to estimate the potential of the probability density function, the observed intensity value is retained. Then, the probability of the new intensity value is calculated by the function. 

### 2.3. Dynamic Background Modeling

In many video analysis applications, detecting moving objects from a video sequence captured by video sensor is one of the basic works. A common approach for this work is background modeling and subtraction, which first builds a suitable background model, and then the moving object will be extracted and labeled as foreground by the approach named background subtraction. This work of detection of moving objects is difficult to accomplish with the purpose of very high accuracy. The performance of background subtraction depends mainly on the background modeling technique used.

Pixel-based methods [51,52] assume that the time series of observations is independent of each pixel, which is unreasonable and restricts their use in dynamic background. In contrast, there are also some region based methods [53,54]. Similar to the textured-based method used in [55,56], it modeled the background with a group of histograms based on local binary patterns (LBP). This method to some extent can avoid labeling some moving background pixels as foreground since it extracts region texture features, which means it can be applied to the dynamic background scenario.

LBP algorithm has good performance during sudden changes in light conditions, LBP texture feature for maintaining robust background and foreground can be well positioned location and outline proposed moving object detection algorithm for robust to changes in illumination. Heikkila et al. [55,57] have done much research on the LBP algorithm including the region based method and the representation of LBP feature on a circular region. Since the LBP algorithm has some advantages, researchers in this filed have developed many impressive embranchments of it. Zhao et al. [58] have proposed an method called VLBP (Volume Local Binary Patterns) which has two drawbacks: (1) it can only be used offline because of the requirement that the whole video sequence must be acquired before computing; and (2) it considers too many pixels lead to a time-consuming counting process. Zhang et al. [56] have proposed an algorithm named STLBP (Spatio-Temporal Local Binary Patterns).

## 3. Moving Object Detection

**Figure 1:**
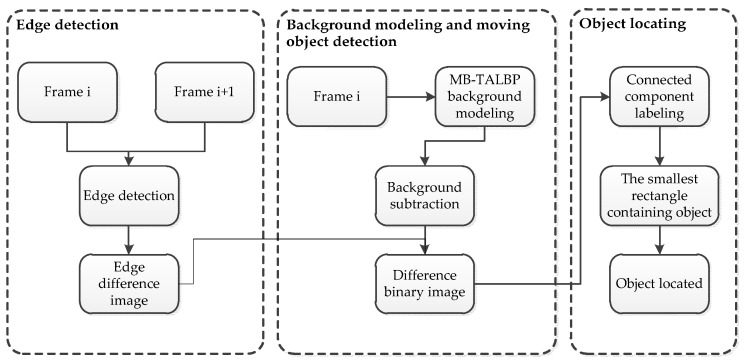
The flow chart of the proposed scheme.

In this section, we present the proposed procedure for moving object detection. To achieve better detection than previous standalone approaches, our proposed scheme uses both background subtraction and edge detection methods simultaneously. Our method is able to extract abundant information from the object both inside and along the edge by using two detection methods (see Figure 1).

Two consecutive frames are used to generate an edge difference image. The concrete steps are: first, do the edge detection on each of the images (which can generate two edge detected images), then do the frame difference with those two images that will generate a binary edge difference image. Our background modeling method (MB-TALBP) is used to generate background image and do the background subtraction with the current frame. After the step of background subtraction, we can also gain a binary image of the foreground. We let both of these two binary images do logic “AND” operation to generate a final difference image, which is the one we use to locate the object.

### 3.1. Moving Edge Detection

Moving edge is the edge of moving objects. Difference image can be regarded as time gradient, while edge image is space gradient. Moving edge can be defined by the logic AND operation of difference image and the edge image [59]. The advantage of frame difference method is its small calculation, and the disadvantage is that it is sensitive to noise, for which another procedure we propose, background subtraction, can provide a partial solution. If the objects do not move but the brightness of the background changes, the results of frame difference methods may not be accurate enough. In order to avoid the weakness (sensitive to the illumination) and enhance its advantage (small calculation), we use the edge detection method to do the same part of work, which can overcome the disadvantage of frame difference method.

In this paper, we use canny operator to do the edge detection. Overall, the edge detection of the image must meet two steps: first, effectively suppress noise, by Gaussian operator image smoothing; and second, try to determine the exact position of the edge. Canny operator edge detection can be divided into four steps: (1) Gaussian smoothing function for the purpose of smoothing to remove noise; (2) first difference convolution template, to achieve edge enhancement; (3) non-maxima suppression (NMS), intended to preserve the maximum gradient direction (The third one is the most important step in this process); and (4) bilinear threshold, to get the edge points in order to determine whether the second step of its 80 neighborhood is obtained, and then make the connection.

### 3.2. Background Modeling

In this paper, we use LBP operator to do the background modeling. There are several advantages of this operator. First, when come into those complex scenarios, such as swaying trees, undulating lake, and flashing monitor, during these situations, in the background there is a new object that enters or older objects are removed, there is a good adaptability. Second, comparing to other common algorithm, such as GMM, this method cause less computational time. Third, this operator also has a good inhibition for noise.

Although the LBP operator has a good performance on background modeling, there are still some work that cannot be accomplished appropriately. We use an improved LBP operator, which combined with “multi-scale block LBP” [60] and “Spatio-temporal LBP” [56], do the modeling work so that the motion and texture information on space and time is on the binding. The computation is done based on average values of block sub regions, instead of individual pixels. In this way, the method presents several advantages: (1) it not only contains the connection between microstructures but also macrostructures of the image being processed, and then provides a more complete image representation than the basic LBP operator; and (2) this method can be computed very efficiently using integral images. Furthermore, in order to reflect the uniform appearance of LBP, we redefine the uniform patterns via statistical analysis.

While using LBP operator, we confirm the size of our operator, which, in the basic method, is 3 × 3. In this paper, we use lager operator to get some more information from the macrostructures, whose size is 9 × 9. This operator is composed of nine 3 × 3 pixel blocks. In each sub-region, average sum of image intensity is computed. These average sums are then compared with the gray value of the center block. After that, the LBP method is used to do the background modeling work. 

We use the following equation to do the comparing work between the center pixel and the pixels from its neighborhood, which is the basic equation of a LBP modeling method.

(1) $$L\left(x_{c},y_{c}\right)=\sum_{p=0}^{7}s(g_{p}-g_{c})2^{p}$$

where $g_{c}$ corresponds to the grey value of the central pixel $\left(x_{c},y_{c}\right)$ and $g_{p}$ to the grey values of the eight neighboring pixels. p is the number of neighboring pixels, which is 8 here. The function s(x) is defined as follows:

(2) $$s\left(x\right)=\left\{ \begin{array}{l} 1\;x\geq 0\\ 0\;x<0 \end{array}\right.$$

The result of computing this equation is counted as the LBP feature of one pixel or block, which is the center part of the operator. LBP features are robust to monotonic gray value changes and very fast to compute, which are very important for background modeling and subtraction. As mentioned above, achieving “real-time” requirement is of great importance to our system.

**Figure 2:**
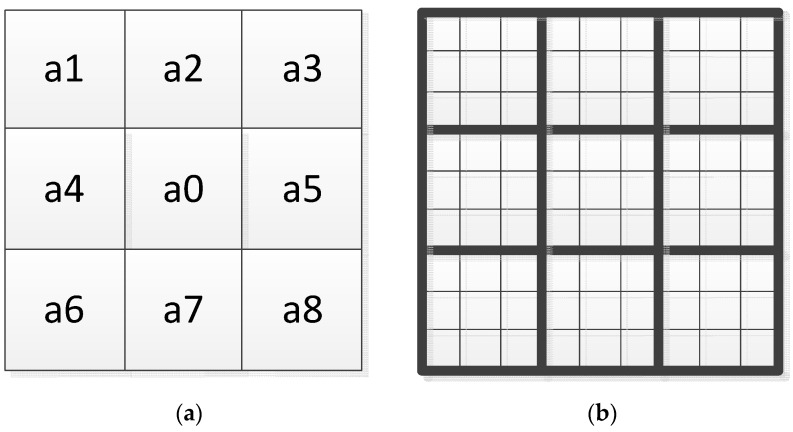
(**a**) The basic 3 × 3 operator of the LBP algorithm; and (**b**) multi-scale LBP (Local Binary Pattern) based on blocks of 9 × 9 size. Computation is done based on average values of block sub regions, instead of individual pixels. In each sub-region, average sum of image intensity is computed. These average sums are then compared with the gray value of the center block.

The form of the LBP operators are shown in Figure 2; the 9 × 9 sized blocks consist of many 3 × 3 blocks. The 3 × 3 blocks are the base unit of all the different sized operators and the size of the operators can be 3 × 3, 9 × 9, and even 27 × 27. On the basis of our research, the operators that are larger than 9 × 9 are not very helpful to the background modeling work. For these reasons, we only use 3 × 3 and 9 × 9 sized operators to do the background modeling work. After using this expended LBP operator and obtaining the coded feature of all the blocks of the processing image, we can integrate the entire feature into a histogram that describes the background feature of this scenario. The comparison between two images is processed by histogram intersection [61]. The equation below shows how the histogram intersection works.

(3) $$\cap \left(a,b\right)=\sum_{n=0}^{N-1}\min\left(a_{n},b_{n}\right)$$

where “*a*” and “*b*” are the histograms generated by 3 × 3 and 9 × 9 operators, respectively. *N* is the number of histogram dimensions. This measure has an intuitive motivation in that it calculates the common part of two histograms. Its advantage is that it explicitly neglects features that only occur in one of the histograms. The complexity is linear for the number of histogram dimensions. It is also possible to use other measures but this histogram intersection method is a more practical and efficient one.

After computing the single frame data of the gained video, the connection between two continuous frames are embodied by the method that we named as “temporal-analysis LBP”. The equations of this algorithm are as follows:

(4) $$L_{P,R}^{t}\left(x_{t,c},\;y_{t,c}\right)=\sum_{p=0}^{P-1}s\left(g_{t,p}-g_{t,c}\right)2^{P}$$

(5) $$L_{P,R}^{t-1}\left(x_{t-1,c},\;y_{t-1,c}\right)=\sum_{p=0}^{P-1}s\left(g_{t-1,p}-g_{t-1,c}\right)2^{P}$$

$\left(x_{t,c},\;y_{t,c}\right)$ and $\left(x_{t-1,c},y_{t-1,c}\right)$ are the central pixel of current and previous frames. $L_{P,R}^{t}\left(x_{t,c},\;y_{t,c}\right)$ and $L_{P,R}^{t-1}\left(x_{t-1,c},\;y_{t-1,c}\right)$ are the spatial and temporal local binary patterns of pixel located in $\left(x_{t,c},\;y_{t,c}\right)$ and $\left(x_{t-1,c},y_{t-1,c}\right)$. *R* is the label of the size of the LBP blocks (which is “3, 9” here), and is not involved in computations. The former extracts the spatial texture features and the latter extracts the motion information of the neighboring two frames. We can compute two histograms $H_{t}$ and $H_{t-1}$ over this region. The equation to compute those two histograms are shown below.

(6) $$H_{t}=\omega H_{t-1,i}+\left(1-\omega \right)H_{t,i},\;i=0,...,2^{P}-1$$

The equation above shows the summing operation between two bins corresponding to each histogram. The parameter “ω” of this temporal-analysis method are determined by the influence the previous frame exerts on the current frame, which we defined elementarily as 0.5. 

**Figure 3:**
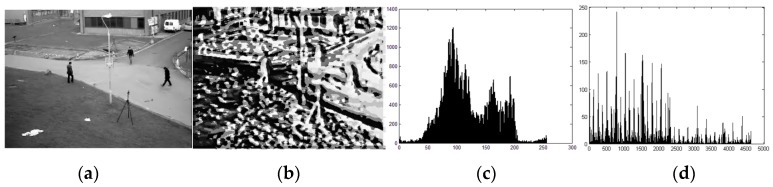
(**a**) The gray scale image of the original image; (**b**) the texture image of the background; (**c**) the histogram of the original image; and (**d**) the LBP histogram of the background image, which shows extensive information of the texture.

Figure 3 show the image processed by LBP algorithm, which embodied rich texture information.

### 3.3. Object Locating

After the background modeling work has been done, we can easily extract the moving object from the frames. Although the object has been extracted, we still need to label the object by a rectangle for visual display. In this paper, we use connected component labeling to do the object locating work.

This process is to label the connected components in binary images, delete those connected components whose area are too small (false positives and noise), and get rounding rectangle of the object. In this section, our method is the following steps:

1. Progressive scan the images: Put each line in a continuous white pixels form a sequence called a “group”, and note down its starting point, end point and the line number.
2. Except for the first row of all the rows: If it has no overlap area with the previous row, then give this row a new tab; if it has only one overlap area with previous row, then give it the same tab with the above one; if it has two or more overlap areas with the above area, then give it the smallest number of those areas and note down all these groups into an equivalent pair, indicating that they belong to the same class.
3. Convert the equivalent pair to equivalent sequences: Give each sequence the same reference numeral, since it is equivalent. Starting from Step 1, we give each equivalent sequence a tab.
4. Traverse the groups from the beginning one: Find each equivalent sequence, and give it a new tab.
5. Fill the label of each group in the marked image.

Once these steps have been done, we can get every eligible labeled area, meaning we can finally locate the object extracted from the image.

## 4. Experiment and Analysis

In order to evaluate our method and compare it with other algorithms, we conduct this experiment using some video sequences. In this section, we first introduce our experiment setup in Section 4.1, including the detailed introduction of the hardware platform we used and the “high-precision intelligent holder” that we mentioned in the title of this paper. We then show the result of software simulation using those result images in Section 4.2, which is for a visual comparison. Finally, the quantitative analyses of our experiments are shown in Section 4.3 by utilizing several tables.

### 4.1. Experimental Setup

To prove the ability of our method in background subtraction step, we mainly focus on the performance of detection methods on situations lacking training opportunities, including situations with illumination change and a dynamic background. In the experiments, we first use the video sequences to test the ability of each method in dealing with situations, such as those mentioned above. We then use the sequences to verify the capability of each method in coping with those situations. First, we select five test video sequences, each moving objects in its initial frames and the continuous flow of moving objects throughout the sequence. 

**Figure 4:**
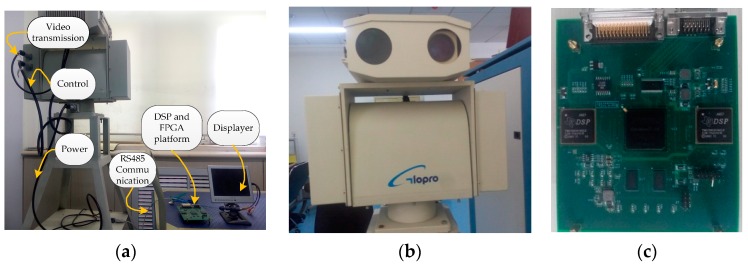
(**a**) Our moving object detecting system and the description of each component; (**b**) the high-precision intelligent holder; and (**c**) the platform of FPGA (Field Programmable Gate Array) and DSP (Digital Signal Processor).

The hardware we put in use is combined with a camera, a platform with FPGA and DSP, a high-precision intelligent holder. All of our hardware platform components interact with each other and accomplish the detection work together. We show each of the portions in Figure 4 with some material images that will make a visual introduction of the whole system. The camera is treated as a main video sensor. 

#### 4.1.1. Methods for Comparison

In this section, we introduce the methods we used to compare with our algorithm below:

1. GMM (Gaussian Mixture Modeling) is a background modeling based approach, which is based on each pixel in the time domain to build the distribution model of each pixel sequentially to achieve the background modeling purposes [36,37,38]. Gaussian mixture background model is a weighted finite number of Gaussian functions which can describe the state of the pixel multimodal, suitable for light gradient, swaying trees and other complex background accurately modeled. Through continuous improvement of many researchers, the method has become the most common background extraction method.
2. ViBe (Visual Background extractor) is a high-efficiency algorithm. This algorithm adopts a new thought to detect targets using random principles in the object detection work. The basic idea is, for each pixel, random sampling radius R within the scope of the model as a background pixel, and the default is 20 sampling points. Compared to some other detection algorithms, ViBe has a small amount of calculation, small footprint, fast processing speed, good detection effect, faster speed and the ablation area of Ghost stable and reliable characteristics respond noise.
3. GMG (an algorithm for finding the Global Minimum with a Guarantee) combines the static background image and each pixel Bayesian estimation division. It uses very little information before (the default is 120 before) the image background modeling. It uses probability prospects estimation algorithm (using a Bayesian estimation to identify prospects). This is an adaptive estimation, the new observed objects have more weight than the old object, which means the results adapt to light changes. Some morphological operations such as opening operation, closing operation, etc. are used to remove unwanted noise.
4. KDE (Kernel Density Estimation) is a well-known moving object detection algorithm. By employing a few frames of the method data, the algorithm can do background modeling with a fast extraction of moving targets in subsequent frames. However, the noise is large and some small moving objects are easily lost. The model is based on estimating the intensity density directly from sample history values. The main feature of the model is that it represents a very recent model of the scene and adapts to changes quickly. A framework was presented to combine a short-term and a long-term model to achieve more robust detection results [49,50].
5. LBAdaptiveSOM (Local Background Adaptive Self-Organizing Modeling) [62] is a self-organizing method for modeling background by learning motion patterns and so allowing foreground/background separation for scenes from stationary cameras. The method is strongly required in video surveillance systems. This method learns background motion trajectories in a self-organizing manner, which makes the neural network structure much simpler. The approach is suitable to be adopted in a layered framework, where operating at region-level, it can improve detection results allowing to more efficiently tackle the camouflage problem and to distinguish moving objects from those that were initially moving and have stopped.

#### 4.1.2. Test Video Sequences

We choose some video sequences from the BMC (Background Models Challenge) dataset including dynamic background, illumination variation, introduction or removal of background objects, noise-corrupted, etc. To be specific, we select five typical crowded scenarios from the BMC (Background Models Challenge) [63] (including a campus, a road, a railway station, a night scenario and a parking lot). For each selected indoor sequence coming from the BMC dataset, manually-labeled ground-truth references are provided. We choose five video sequences of this benchmark dataset to show the visual comparisons in Section 4.2 (each video show one typical frame); after that, we will show the quantitative comparisons in Section 4.3 (experimental data are from the whole sequences). All of the test data are provided with manually-labeled ground-truth references.

### 4.2. Visual Comparisons

All of the videos we show in this section are from the BMC dataset. Based on the consideration of clarity and representation, we choose five video sequences in the dataset to show the results of these comparative experiments. The experiment results of all five shown video sequences and the rest will be presented in line chart form in the analyzing portion in Section 4.3. 

According to the order of appearance, they are: “Wandering students”, “Rabbit in night”, “Beware of the trains”, “One rainy hour” and “Big trucks”.

**Figure 5:**
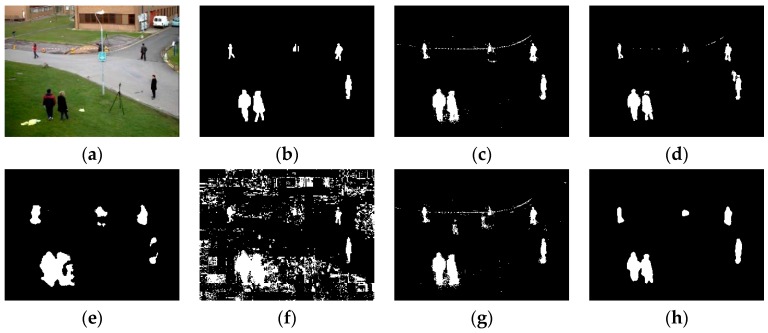
(**a**) Frame 555 of input images; (**b**) the ground truth; (**c**) GMM (Gaussian Mixture Modeling); (**d**) ViBe (Visual Background extractor); (**e**) GMG (an algorithm for finding the Global Minimum with a Guarantee); (**f**) KDE (Kernel Density Estimation); (**g**) LBAdaptiveSOM (Local Background Adaptive Self-Organizing Modeling); and (**h**) our method.

**Figure 6:**
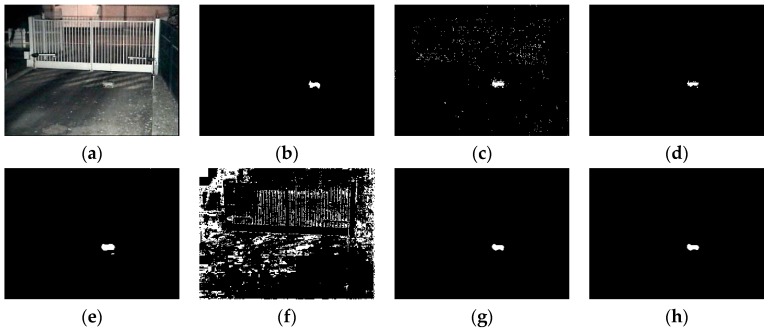
(**a**) Frame 1252 of input images; (**b**) ground truth; (**c**) GMM (Gaussian Mixture Modeling); (**d**) ViBe (Visual Background extractor); (**e**) GMG (an algorithm for finding the Global Minimum with a Guarantee); (**f**) KDE (Kernel Density Estimation); (**g**) LBAdaptiveSOM (Local Background Adaptive Self-Organizing Modeling) ; and (**h**) our method.

**Figure 7:**
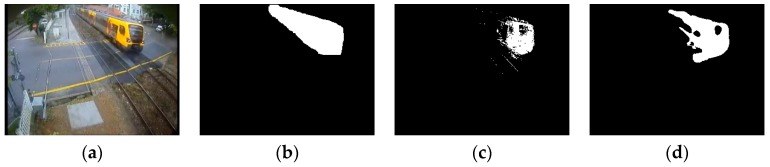

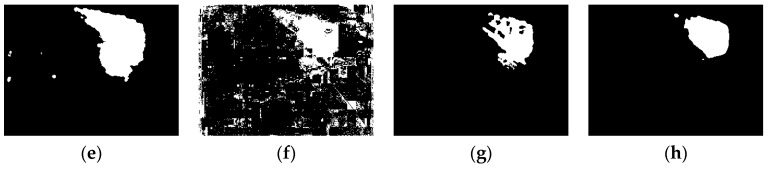
(**a**) is the frame 389 of input images; (**b**) ground truth; (**c**) GMM (Gaussian Mixture Modeling); (**d**) ViBe (Visual Background extractor); (**e**) GMG (an algorithm for finding the Global Minimum with a Guarantee); (**f**) KDE (Kernel Density Estimation); (**g**) LBAdaptiveSOM (Local Background Adaptive Self-Organizing Modeling); and (**h**) our method.

**Figure 8:**
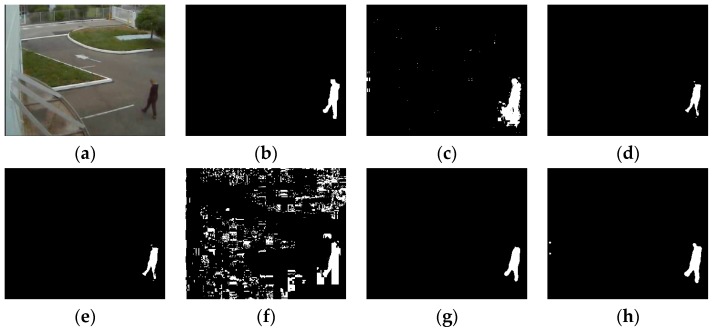
(**a**) is the frame 15553 of input images; (**b**) ground truth; (**c**) GMM (Gaussian Mixture Modeling); (**d**) ViBe (Visual Background extractor); (**e**) GMG (an algorithm for finding the Global Minimum with a Guarantee); (**f**) KDE (Kernel Density Estimation); (**g**) LBAdaptiveSOM (Local Background Adaptive Self-Organizing Modeling); (**h**) our method.

**Figure 9:**
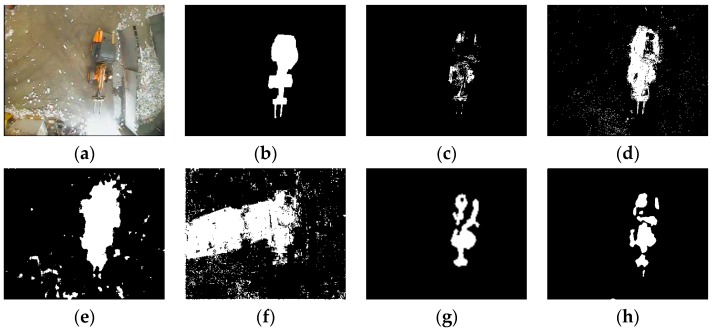
(**a**) is the frame 485 of input images; (**b**) ground truth; (**c**) GMM (Gaussian Mixture Modeling); (**d**) ViBe (Visual Background extractor); (**e**) GMG (an algorithm for finding the Global Minimum with a Guarantee); (**f**) KDE (Kernel Density Estimation); (**g**) LBAdaptiveSOM (Local Background Adaptive Self-Organizing Modeling); (**h**) our method.

Some representative experimental results are shown in Figure 5, Figure 6, Figure 7, Figure 8 and Figure 9, the result of KDE algorithm have quite low metrics, because of its poor ability to resist noise disturbance that other methods do not have. As a kind of classic algorithm, GMM algorithm gives some stable but ordinary results. ViBe algorithm, which has fast calculation speed, has similar results with GMM. GMG can generate a good result in some scenarios but in other situations the result can be hard to accept. Through a visual judgment of those images we show in this section, our method always gets the best results.

Some of the videos selected have a dynamic background, such as “Wandering students” and “One rainy hour”; and some of them have illumination variations, such as “Rabbit in night”. When processing a video with dynamic background, some of the algorithms do not perform well. As we can see, in the first sequence, GMM, ViBe, and KDE all regard the dynamic part of background as a detected object and the same situation also shows in other video sequences. Illumination variations also produce some influence of those methods. Similar to the results of the second sequence (“Rabbit in the night”), GMM, GMG, and KDE cannot generate the full object.

### 4.3. Quantitative Comparisons

To assess the detection results objectively, four indexes (i.e., Recall, Precision, F-measure and Similarity) are put to use by us to evaluate the performance of these techniques at the pixel level. Among these four, TP is the number of true positives, TN represents the number of true negatives, FP means the number of false positives, and FN represents the number of false negatives. The four indexes are defined as:

(7) Recall=TPTP+FN

(8) $$\mathrm{Precision}\;=\frac{\mathrm{TP}}{\mathrm{TP}+\mathrm{FP}}$$

(9) $$\mathrm{F-means}=\frac{\left(\alpha^{2}+1\right)\mathrm{PR}}{\alpha^{2}\left(P+R\right)}$$

(10) $$\mathrm{Similarity}\;=\frac{\mathrm{TP}}{\mathrm{TP}+\mathrm{FN}+\mathrm{FP}}$$

We can discover something that the two indexes “Recall” (R) and “Precision” (P) a contradict each other. When this happens, we use the latter two indexes to achieve an overall consideration. When α = 1, $\mathrm{F-means}\;=\;F-1=\;\frac{2\mathrm{PR}}{P\;+\;R}$.

In this section, we mainly do a quantitative comparison of six different moving object detection methods with our method. All the video sequences are the same as the ones used in the previous section for visual comparisons.

We made several tables (Table 1, Table 2, Table 3, Table 4 and Table 5) to provide a more intuitive data form. Each table represents a given video sequence from the dataset.

We employ some tables to show the comparison of the indexes mentioned in this section. The tables show the metrics for one typical frame in each video sequence. Clearly, the results of KDE algorithm have quite low metrics, because of its poor ability to resist noise disturbance; and GMM, ViBe, GMG, LBAdaptiveSOM and our method (we call it MB-TALBP) produce much better results in this respect, and MB-TALBP ranks first in the index “Precision” for all videos. Compared to all of the indicators (i.e., F-1 and Similarity), our method performs the best.

The first video has a dynamic background. As shown in Table 1, our method has good performance in this scenario. The other methods in this experiment do not perform very well because these algorithms regard the dynamic background as foreground. This experiment shows the advantage we have in the dynamic background situation.

This video, named “RABBIT IN THE NIGHT”, has an illumination change. From the table, we can see that our method has a large advantage compare to the other methods. That is to say, our method can deal with the situation of illumination change very well, which is a significant advantage.

The two video sequences above both have strong video noise. From the result of the experiment, our method has a good result, better than the other methods. These two experiment results show that the proposed method has good immunity to video noise.

The fifth video in this experiment has a frequently changing background, which is a challenge for every algorithm. Most algorithms get poor result in this condition, as shown in Table 5. ViBe has a fast speed of background modeling, which is a great advantage when dealing with this situation. Although the experiment result of our method is not as good as ViBe in this condition, our method still performs better than the other methods. In addition, according to the experiment data, the gap between our method and ViBe algorithm is not too large. The proposed method still gets second place in this experiment.

As shown in Table 1, Table 2, Table 3, Table 4 and Table 5, our method (MB-TALBP) has the best results in four of five experiments. In Table 1 and Table 4, since there is an obvious dynamic part in the backgrounds of the test video sequences, GMM, GMG, KDE and ViBe methods perform poorly. The LBAdaptiveSOM method shows a better result compared to those four algorithms, and a little worse than our method. Table 2 shows the experiment results of the sequence named “rabbit in night”, which has an illumination change scenario. As shown in Table 2, our method still performs the best in this complex scenario. In Table 3, the backgrounds of the test sequences changes quickly, and our method performs better than four methods and a little worse than ViBe. The fifth video sequence has the frequent introduction and removal of objects and the indexes in Table 5 show that our method has the second best result, which means our method, is stable when facing this complex situation. The fifth video sequence has a frequently changed background and most methods in this experiment have a low result. ViBe algorithm has a fast speed of background modeling and is really good at dealing with the situation in video 5, as seen in Table 5. Although the ViBe algorithm has a better result in one of the experiments, our method still has the advantage in the rest of the experiments, which means our method performs better than the ViBe algorithm.

**Figure 10:**
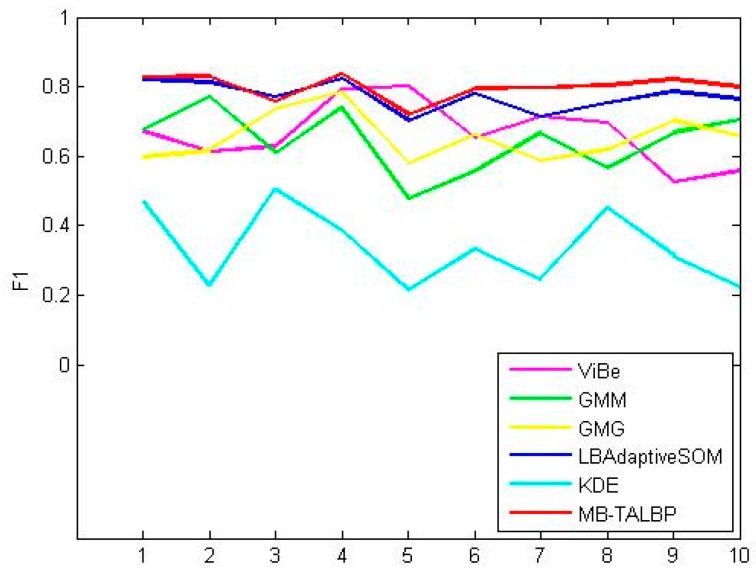
This image shows the line chart of the F-1 value of the experiment result of those methods we mentioned above.

Our method still has the second best results in the experiments in which our method is not the best one. That is to say, our method can adapt to dynamic backgrounds, illumination variations, noise-corruption and other complex scenarios very well. Figure 10 shows the experiment results of all the 10 videos.

The quantitative comparisons have indicated that our method has a good advantage over the other methods in real scenes that often occur in reality and is could be of great use when applied to those situations that require rapid and accurate detection of moving objects. Our method has been evaluated against five methods in 10 different video sequences including dynamic background, illumination variation, introduction/removal of background objects and noise-corrupted scenes. Figure 7 shows that our method performs well in most experiments and comparisons to other approaches presented in this experiment have shown that our approach has an obvious advantage to four of the methods (GMM, ViBe, GMG and KDE). It has proven to be tolerant to the dynamic background, and introduction and removal of background objects. Furthermore, the method is capable of dealing with illumination variations. 

The proposed method is implemented on a PC with an i5-4570 3.2-GHz processor and 4 Gb RAM. Our MATLAB algorithm (including showing the binary results) can achieve the processing speeds of 26 fps, 35 fps and so on in the experiment. The detailed data are as follows.

The data of execution time are shown in Table 6: the execution time of our method in these experiments has an average result of 39.92 ms/frame (which is 25.05 fps). The portion of background modeling work (the LBP algorithm) takes most of the computation time, which means the execution time of background modeling determines the whole execution time. From these data, we can see our method has achieved the real-time requirement. 

The hardware platform we used has one EP3C120F780I7N FPGA chip and two TMS320C6416 DSP chips as its core control and processing units, respectively. In our research, the execution time of processing one frame on the hardware platform is 49–54 ms (which is 18–20 fps). The speed of data processing on the hardware platform can also achieve the real-time requirement.

## 5. Conclusions

In this paper, we proposed a combinational method for moving object detection. In our method, the multi block temporal-analyzing LBP algorithm is used to do the background modeling work. Additionally, the edge detection method is only applied to enhance the ability of resisting illumination variation. The connected component labeling method is used to locate the object from the segmentation result. Experimental results show the preponderance of the proposed method in terms of precision, which is an important role of moving object detection. Though our method can handle illumination variation, dynamic background and video noise well, there are still some situations that it cannot handle very well, such as the frequently changing of background. Further research should be proceed from this angle: dealing with scenarios that have frequently changing backgrounds. The speed of background modeling, the frequency of background updating and the simplification of computing the background model are the primary issues that should be considered in our future research. We believe that solving these problems will enhance our method. 

## Figures and Tables

**Table 1:** Comparison of indexes of “Wandering students”.

Method	Precision	Recall	F-1	Similarity
ViBe	0.7452	0.6134	0.6729	0.4966
GMM	0.7538	0.6085	0.6734	0.4801
GMG	0.8801	0.4521	0.5973	0.4007
LBAdaptiveSOM	0.9426	0.7259	0.8202	0.6847
KDE	0.5112	0.5438	0.4681	0.3274
MB-TALBP	0.9521	0.7372	0.8279	0.7142

**Table 2:** Comparison of indexes of “Rabbit in the night”.

Method	Precision	Recall	F-1	Similarity
ViBe	0.7678	0.8887	0.6130	0.4419
GMM	0.7121	0.8431	0.7721	0.6542
GMG	0.8546	0.4781	0.6159	0.4788
LBAdaptiveSOM	0.9216	0.7249	0.8115	0.6630
KDE	0.3325	0.1716	0.2263	0.1156
MB-TALBP	0.9332	0.7470	0.8298	0.7049

**Table 3:** Comparison of indexes of “Beware of the trains”.

Method	Precision	Recall	F-1	Similarity
ViBe	0.6949	0.5100	0.6286	0.5270
GMM	0.7051	0.5349	0.6083	0.4412
GMG	0.8325	0.6591	0.7357	0.5869
LBAdaptiveSOM	0.8547	0.7026	0.7712	0.5940
KDE	0.5465	0.4692	0.5049	0.3323
MB-TALBP	0.8456	0.6914	0.7807	0.6912

**Table 4:** Comparison of indexes of “One rainy hour”.

Method	Precision	Recall	F-1	Similarity
ViBe	0.8288	0.7600	0.7929	0.6494
GMM	0.8546	0.6527	0.7401	0.6095
GMG	0.8167	0.7533	0.7837	0.6681
LBAdaptiveSOM	0.9254	0.7441	0.8232	0.7354
KDE	0.4267	0.3491	0.3840	0.1729
MB-TALBP	0.9400	0.7529	0.8361	0.7456

**Table 5:** Comparison of indexes of “Big trucks”.

Method	Precision	Recall	F-1	Similarity
ViBe	0.9024	0.7195	0.8006	0.6819
GMM	0.5726	0.4117	0.4764	0.2202
GMG	0.7649	0.4674	0.5802	0.3519
LBAdaptiveSOM	0.7752	0.6429	0.7029	0.5461
KDE	0.1438	0.4194	0.2142	0.0826
MB-TALBP	0.8053	0.6521	0.7206	0.5583

**Table 6:** Execution time of MB-TALBP (Multi-Block Temporal-Analyzing Local Binary Pattern) in software experiments.

Video	1	2	3	4	5	6	7	8	9	10
Time (ms/frame)	42.3	41.7	43.0	27.6	40.6	40.8	35.3	43.4	29.8	33.7
